# Changes in the meibomian glands in postmenopausal women with primary acquired nasolacrimal duct obstruction: a prospective study

**DOI:** 10.1186/s12886-023-02799-3

**Published:** 2023-02-01

**Authors:** Haili Jin, Hong Zhang

**Affiliations:** 1 Department of Ophthalmology, Tianjin Medical University, NO. 22, Qixiangtai Road, Tianjin, 300052 China; 2 Department of Ophthalmology, Wuhu Eye Hospital, Anhui University of Science & Technology, 378 Santan Road, Yijiang District, Wuhu, 241002, Anhui China; 3 Department of Ophthalmology, Sino-Singapore Eco-City Hospital of Tianjin Medical University, NO. 3333, Hechang Road, Sino-Singapore Eco-City, Binhai New Area, Tianjin, 3000467 China

**Keywords:** Primary acquired nasolacrimal duct obstruction, Postmenopausal women, Meibomian gland loss

## Abstract

**Background:**

Primary acquired nasolacrimal duct obstruction (PANDO) is frequently encountered in perimenopausal women, causing tear flow stagnation and resulting in a variety of ocular discomfort symptoms. However, little is known about the alterations in the meibomian gland in postmenopausal women with PANDO. Hence, this study investigated the changes in the meibomian gland and ocular surface in postmenopausal women with PANDO.

**Methods:**

This prospective study included 60 eyes of 60 postmenopausal women with PANDO (PANDO group) and 30 eyes of 30 postmenopausal women without PANDO (control group). The PANDO group was further subdivided into incomplete and complete PANDO groups, based on the degree of nasolacrimal duct obstruction. The patients’ symptoms were evaluated using the ocular surface disease index questionnaire. The meibomian gland and ocular surface were assessed using the Keratograph 5 M. Other ophthalmologic examinations included the tear break-up time, corneal fluorescein staining, meibomian gland expression, and Schirmer I test. The correlations between the degree of nasolacrimal duct obstruction and other metrics were analyzed.

**Results:**

The loss ratio of the upper eyelid was greater in the incomplete PANDO group than in the control group (*p* = 0.023). Meibomian gland distortion of the upper eyelid was more severe in the control group than in the complete PANDO group (*p* = 0.022). The non-invasive tear meniscus height was greater, whereas the intensity of corneal fluorescein staining was lower in the PANDO group than in the control group (all *p* < 0.05). The degree of nasolacrimal duct obstruction was positively associated with the non-invasive tear meniscus height and ocular surface disease index scores (*p* < 0.001 and *p* < 0.001, respectively). Corneal fluorescein staining and meibomian gland distortion of the upper eyelid were negatively correlated with the degree of nasolacrimal duct obstruction (*p* = 0.01 and *p* = 0.007, respectively).

**Conclusion:**

Postmenopausal women with PANDO exhibit significant morphological changes in the meibomian gland. More attention should be paid to meibomian gland loss in postmenopausal women with incomplete PANDO, as it is crucial for identifying meibomian gland impairments in patients with PANDO.

## Background

Primary acquired nasolacrimal duct obstruction (PANDO) [1–3] is commonly encountered in ophthalmic practice, especially among perimenopausal women. Chronic inflammation and nasolacrimal duct fibrosis are the principal processes involved in the pathogenesis of PANDO. Tear flow stagnation induced by nasolacrimal duct obstruction (NLDO) is responsible for various manifestations of ocular discomfort such as epiphora and viscous discharge. The incidence of PANDO increases with age [4], making it a significant public health concern, as the global elderly population is predicted to more than double by 2050. Although surgery yields good outcomes, approximately 20% of patients with NLDO develop dry eye after treatment, while their quality of life remains unchanged [5, 6]. Therefore, comprehensive examination of dry eye is essential before treatment for PANDO. The tears of patients with PANDO have higher levels of inflammatory cytokines than those of normal individuals [7, 8]. These inflammatory cytokines may cause meibomian gland dysfunction, since the meibomian gland is exposed to the tear film [9]. Meibomian gland dysfunction is a chronic condition characterized by terminal duct blockage or abnormalities in glandular secretion as well as meibomian gland loss [9]. However, hypoosmolarity in the tears of postmenopausal women with PANDO [10] improves tear hyperosmolarity [11], which may be beneficial to the meibomian gland. This observation has prompted clinicians to identify changes in the meibomian gland and ocular surface in postmenopausal women with PANDO.

Few studies have investigated the effects of PANDO on ocular function. In 2001, Kubo et al. [12] reported thickening of the tear lipid layer after dacryocystorhinostomy. In 2016, Yuksel et al. [10] reported that epiphora caused by PANDO led to tear hypoosmolarity. However, studies on meibomian gland alterations in postmenopausal women with PANDO are scarce. Recently, some studies have observed meibomian gland alterations in ocular and systemic diseases using the Keratograph 5 M (Oculus GmbH, Wetzlar, Germany) [13, 14]. This device facilitates non-invasive and objective examination, as it can observe distinct morphological changes in the meibomian gland using infrared imaging technology with excellent reproducibility and reliability [15].

The identification of ocular surface characteristics in postmenopausal women with PANDO can aid ophthalmologists in determining ocular surface damage, and consequently, facilitate the development of better treatment modalities. Improving ocular surface discomfort and preventing other ocular surface diseases is of immense clinical significance to improve the quality of life. Therefore, this study investigated the changes in the meibomian gland and ocular surface in postmenopausal women with PANDO.

## Participants and methods

Participants were stratified into the incomplete PANDO and complete PANDO groups, according to the degree of nasolacrimal duct obstruction in order to identify changes in the meibomian gland and ocular surface in postmenopausal women with PANDO. The 5 M Keratograph and a slit-lamp microscope were used to evaluate the morphology and function of the meibomian gland. The parameters of the ocular surface, structure, and function of the meibomian gland were analyzed.

The morphological changes in the meibomian gland and ocular surface parameters in postmenopausal women with PANDO were analyzed from the perspective of the degree of nasolacrimal duct obstruction using non-invasive methods. The correlation between the degree of nasolacrimal duct obstruction and ocular surface parameters were also investigated.

### Ethical approval

This prospective study was approved by the Ethics Committee of Wuhu Eye Hospital, which is affiliated with Anhui University of Science & Technology (No. 20210107). The study adhered to the principles of the Declaration of Helsinki. The participants provided written informed consent after receiving an explanation of the nature and potential consequences of the study.

### Participants

This prospective study enrolled 60 eyes of 60 postmenopausal women with PANDO (PANDO group; mean age, 61.1 ± 8.4 years) and 30 eyes of 30 postmenopausal women without PANDO (control group; mean age, 62.8 ± 7.75 years) who visited Wuhu Eye Hospital between March 2021 and April 2022. The mean duration of menopause in the PANDO group was 5.36 ± 7.50 years (range, 0.1–20 years). Patients with PANDO were further subdivided into the incomplete PANDO (group A) and complete PANDO (group B) groups based on the degree of lacrimal duct obstruction. The affected eye was chosen as the study eye in the PANDO group, and data from the right eye were analyzed if both eyes were affected. The right eye was used as the study eye for the control group. The diagnosis of NLDO was based on lacrimal irrigation and dacryocystography. Participants with complete NLDO demonstrated complete reflux during syringing, while those with incomplete NLDO demonstrated partial reflux, which was further confirmed by dacryocystography. The degree of lacrimal duct obstruction was classified as mild or severe; partial lacrimal duct obstruction was classified as mild, whereas complete lacrimal duct obstruction was classified as severe. The control group comprised individuals with a normally functioning lacrimal duct system without reflux during syringing, as confirmed in the outpatient clinic, who visited the hospital for vision impairment due to cataract. The exclusion criteria included premenopausal women, history of ocular trauma or surgery, ocular inflammation, diabetes mellitus, continuous use of eye drops or other treatments, long-term contact lens wear, Sjögren’s syndrome, or systemic diseases affecting tear film quality and stability.

### Examinations

All measurements were obtained between 9 and 11 a.m. to eliminate the effect of diurnal variation. The ambient conditions of the examination room were kept relatively constant, with temperatures ranging from 22–28 °C and a relative humidity of 40–50%. The following examinations were performed sequentially: ocular surface disease index questionnaire, Keratograph 5 M examination, tear break-up time, corneal fluorescein staining, eyelid margins, meibomian gland orifices, meibomian gland secretion expressibility, meibum quality, and Schirmer I test.

#### Ocular surface disease index questionnaire

The ocular surface disease index questionnaire was used to evaluate the symptoms of ocular discomfort. The total questionnaire score ranges from 0 to 100, with higher scores indicating greater severity of ocular discomfort [16].

#### Keratograph 5 M examination

The Keratograph 5 M was used for non-invasive measurement of the tear film break-up time, tear meniscus height, lipid layer grading, and infrared meibography. An non-invasive tear meniscus height ≥ 0.2 mm was designated as normal and an non-invasive tear meniscus height < 0.2 mm was designated as dry eye. The non-invasive break-up time of tear film consisted of the first non-invasive break-up time of tear film and average non-invasive break-up time of tear film. The level of the non-invasive break-up time of tear film was classified as follows: normal (first non-invasive break-up time of tear film ≥ 10 s and average non-invasive break-up time of tear film ≥ 14 s) and dry eye (first non-invasive break-up time of tear film ≤ 5 s or average non-invasive break-up time of tear film ≤ 6 s). The lipid layer was graded as follows: level 1 (gray colored with a thin and fuzzy structure), level 2 (normal with a clear structure and rich color), and level 3 (thick with a highly clear structure and rich color) [13]. Following eyelid eversion, infrared images of the meibomian glands were acquired, and Image J version 2.0.0 software (National Institutes of Health, Bethesda, ML, USA) was used to compute the loss of the meibomian gland as the area percentage ratio of the lost portions to the total area of the upper and lower eyelids [13, 17, 18]. Furthermore, meibomian gland distortion was characterized as distortion > 45° in at least one meibomian gland in the upper or lower eyelid. The meibomian gland distortion score ranged from 0 to 2: 0 indicated no distortion, 1 indicated distortion of 1–4 meibomian glands; and 2 indicated distortion of more than 5 meibomian glands [14].

#### Tear break-up time and corneal fluorescein staining

Tear break-up time was measured using fluorescein sodium test strips (Tianjin Jingming New Technology Development Co. Ltd., China). The average of the three tear break-up time measurements was obtained for analysis [19]. A tear break-up time < 5 s is typically associated with dry eye [20]. The total corneal fluorescein staining score was the sum of the scores of the four corneal zones [21] (superior nasal, inferior nasal, superior temporal, and inferior temporal), which were graded individually as 0 (no staining), 1 (mild with 1–30 dots of staining), 2 (moderate staining between grades 1 and 3), or 3 (severe staining with confluent stains, filaments, or ulcers).

#### Eyelid margin assessment

Eyelid margins abnormalities [22] were evaluated using a slit-lamp diffused light for the following abnormal signs: irregular eyelid margin, vascular engorgement, obstructed glandular orifices, and anterior or posterior mucocutaneous junction displacement. If none of the above abnormalities was detected, a score of 0 was assigned. One point was awarded for each finding to obtain a total of 0–4 points.

#### Meibomian gland orifice

The meibomian gland orifices (including capping, narrowing, cuffing loss, obliteration level, opaque or scarred, and pouting) was observed and graded as follows: 0, normal; 1, fat cap; 2, obstruction or stenosis, protuberance; 3, serious obstruction or atrophy.

#### Meibomian gland expression assessment

Meibomian gland expression was examined using slit-lamp biomicroscopy. meibomian gland secretion expressibility was evaluated by applying digital pressure over 5 meibomian glands at the center of the upper eyelid [23]. The number of meibomian glands from which meibum could be expressed was scored from 0 to 3: 0, all 5 meibomian glands; 1, 3–4 meibomian glands; 2, 1–2 meibomian glands; and 3, 0 meibomian glands. Meibum quality [24] of the eight central meibomian glands in the upper and lower eyelid was assessed and scored on a scale of 0 to 3: 0, clear; 1, cloudy; 2, cloudy with debris; and 3, inspissated.

#### Schirmer I test

The secretory function of the main lacrimal gland was evaluated using the Schirmer I test without anesthesia. A value of 5 mm is considered abnormal [25].

### Statistical analysis

Statistical analysis was performed using the SPSS software package (version 22.0; SPSS Inc., Chicago, IL, USA). The normality of data distribution was assessed using the Shapiro–Wilk test. Non-normally distributed variables were analyzed using non-parametric tests. The non-parametric Mann–Whitney U test was used to compare two groups. The non-parametric Kruskal–Wallis H-test was used to analyze variables obtained from the three groups. Subsequently, Spearman’s correlation was used to analyze the relationships between the variables. Statistical significance was set at *p* < 0.05.

## Results

### Participants

The age or menopausal duration did not differ significantly among group A, group B, or the control group.((56.5[54.5, 70.00]) years, (58.5[53.00, 70.00]) years, (63.00[57.5, 68.25])years, (*p* = 0.527) and (6.00[2.75, 18.75])years, (11[3.75, 19.25]) years, (15.00[5.50, 23.00]) years, (*p* = 0.347), respectively). Moreover, the menopausal duration did not differ significantly between groups A and B ((2.00[1.00, 4.25])years vs. (4.5[1, 10.00]) years, (*p* = 0.113)).

### Ocular surface disease index and meibomian gland expression combined with eyelid evaluation

**Fig. 1:**
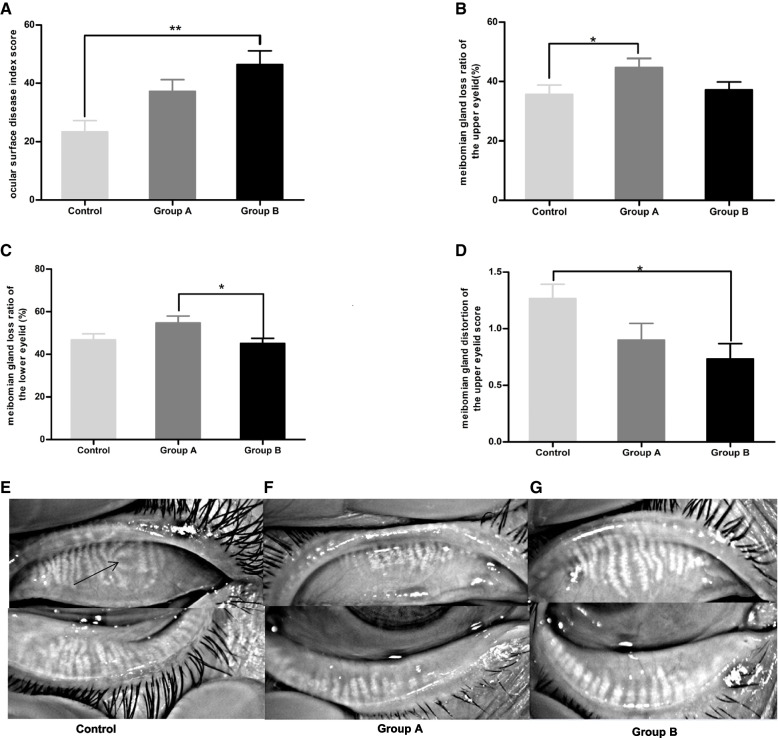
Significant differences in the ocular surface disease index and morphological changes in the meibomian gland among the three groups. The ocular surface disease index scores (**A**) in group B were significantly higher than those in the control group. The meibomian gland loss ratio of the upper eyelid (**B**) in group A was significantly higher than that in the control group. The meibomian gland loss ratio of the lower eyelid (**C**) in group A was significantly higher than that in group B. The meibomian gland distortion of the upper eyelid scores (**D**) in group B was significantly lower than in the control group.*P < 0.05; **P < 0.01; ***P < 0.001. The loss of meibomian gland in control (**E**), group A (**F**), and group B(**G**). The black arrow indicates distortion in the duct

The ocular surface disease index score in group B (50.00[35.21, 66.61]) was significantly higher than that in the control group (22.22[2.68, 36.25]), (*p* = 0.001). The meibomian gland loss ratio of the upper eyelid was greater in group A (42.91[35.64, 56.96]%) than in the control group (32.95[24.26, 42.19]%), (*p* = 0.023). The meibomian gland loss ratio of the lower eyelid was greater in group A (55.17[48.35, 65.62]%) than in group B (43.68[38.02, 53.63]%), (*p* = 0.029). The meibomian gland distortion of the upper eyelid score was significantly lower in group B (1[0,1]) than in the control group (1[1, 2]), (*p* = 0.022). The index values of this study population are presented in Table 1 and Fig. 1.

**Table 1:** Comparison of the Ocular Surface Disease Index score and Meibomian Gland Parameters

	Control (*n* = 30)	Group A (*n* = 30)	Group B (*n* = 30)	*P* value
ocular surface disease index score	22.22[2.68, 36.25]	33.33[24.22, 57.44]	50.00[35.21, 66.61]	0.001
meibomian gland loss ratio of the upper eyelid (%)	32.95[24.26, 42.19]	42.91[35.64, 56.96]	37.03[26.28, 42.31]	0.023
meibomian gland loss ratio of the lower eyelid (%)	45.27[36.72, 56.05]	55.17[48.35, 65.62]	43.68[38.02, 53.63]	0.029
meibomian gland distortion of the upper eyelid (score)	1[1, 2]	1[0, 2]	1[0, 1]	0.022
meibomian gland distortion of the lower eyelid (score)	0[0, 1]	0[0, 0.25]	0[0, 1]	0.208
meibomian gland orifices (score)	1.50[0, 2.00]	1.50[0, 2.00]	2[1, 2.25]	0.087
meibomian gland secretion expressibility (score)	2[1, 3]	2[1, 3]	2[1, 2]	0.664
meibum quality of the upper eyelid (score)	1[1, 1]	1[1, 1]	1[1, 2]	0.214
meibum quality of the lower eyelid (score)	1[1, 1]	1[1, 1]	1[1, 1]	0.962
eyelid margins (score)	2[1, 2.25]	2[2, 3]	2[1, 3]	0.186

The Kruskal–Wallis H-test was performed for the comparison of variables among the three groups

### Stability of tear film, corneal fluorescein staining, and schirmer i test comparison

**Fig. 2:**
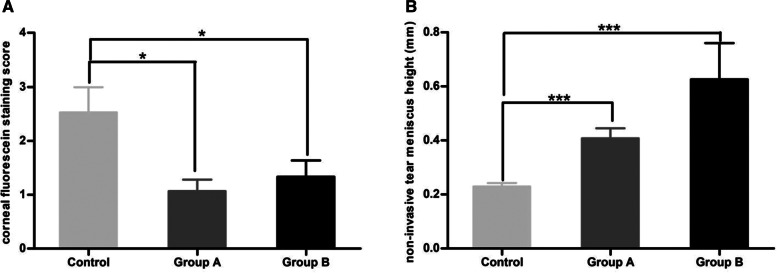
Significant differences in the corneal fluorescein staining score and non-invasive tear meniscus height among the three groups. The corneal fluorescein staining score (**A**) in the control group was significantly higher than that in group A and group B, respectively. The non-invasive tear meniscus height (**B**) in the control group was significantly lower than that in group A and group B, respectively. *P < 0.05; **P < 0.01; ***P < 0.001

The corneal fluorescein staining score of the control group (2[1, 3.25]) was significantly higher than that of group A (1[0, 1.25]),(*p* = 0.015) and group B (1[0, 2]), (*p* = 0.033). The non-invasive tear meniscus height of the control group (0.22[0.19, 0.27]) was significantly lower than that of group A (0.37[0.26, 0.55])mm, (*p* = 0.001) and group B (0.47[0.27, 0.66]) mm, (*p* < 0.001). The index values of this study population are depicted in Table 2 and Fig. 2.

**Table 2:** Comparison of the Ocular Surface Parameters

	Control (*n* = 30)	Group A (*n* = 30)	Group B (*n* = 30)	*P* value
first non-invasive break-up time of tear film (s)	5.16[3.30, 9.17]	8.22[5.11, 17.88]	6.22[3.16, 10.61]	0.098
Average non-invasive break-up time of tear film (s)	8.79[4.90, 14.32]	10.39[5.71, 20.95]	12.37[5.78, 18.35]	0.214
lipid layer grading	2[2, 3]	2[2, 3]	2[2, 3]	0.972
tear break-up time (s)	3.00[2.75, 5.00]	3.00[2.00, 5.25]	3.00[2.00, 5.00]	0.959
Corneal fluorescein staining (score)	2.00[1.00, 3.25]	1.00[0, 1.25]	1.00[0, 2.00]	0.008
non-invasive tear meniscus height (mm)	0.22[0.19, 0.27]	0.37[0.26, 0.55]	0.47[0.27, 0.66]	< 0.001
Schirmer I test (mm)	10.00[5.75, 24.00]	16.50[6.50, 19.00]	14.00[6.00, 22.5]	0.859

The Kruskal–Wallis H-test was performed for the comparison of variables among the three groups

### Correlation analyses between the degree of nasolacrimal duct obstruction and ocular surface parameters

The non-invasive tear meniscus height and ocular surface disease index scores were positively correlated with the degree of nasolacrimal duct obstruction (*R* = 0.492, *p* < 0.001; *R* = 0.381, *p* < 0.001, respectively). The corneal fluorescein staining and meibomian gland distortion of the upper eyelid scores were negatively correlated with the degree of nasolacrimal duct obstruction (*R* = -0.269, *p* = 0.01; *R* = -0.285, *p* = 0.007, respectively). The results of correlation analyses between the degree of nasolacrimal duct obstruction and ocular surface parameters are enumerated in Table 3.

**Table 3:** Correlation Between the Ocular Surface Parameters and the degree of nasolacrimal duct obstruction

	*R*	*P*
ocular surface disease index (score)	0.381^***^	< 0.001
loss ratio of the upper eyelid (%)	0.047	0.663
loss ratio of the lower eyelid (%)	-0.041	0.699
meibomian gland distortion of the upper eyelid (score)	-0.285^**^	0.007
meibomian gland distortion of the lower eyelid (score)	-0.152	0.152
meibomian gland orifices (score)	0.510	0.636
meibomian gland secretion expressibility (score)	0.084	0.432
meibum quality of the upper eyelid (score)	0.166	0.118
meibum quality of the lower eyelid (score)	-0.025	0.817
first non-invasive break-up time of tear film (s)	0.039	0.717
average non-invasive break-up time of tear film (s)	0.158	0.138
non-invasive tear meniscus height (mm)	0.492^***^	< 0.001
lipid layer grading	0.015	0.887
tear break-up time (s)	0.016	0.877
corneal fluorescein staining	-0.269^**^	0.01
Schirmer I test (mm)	0.055	0.608

Spearman’s correlation analysis significance: *P-value < 0.05; **P-value < 0.01; ***P-value < 0.001

## Discussion

Tear hypoosmolarity [15] and pro-inflammatory factor upregulation [7, 8] observed in patients with PANDO can be detrimental to the ocular surface cells, leading to structural alterations and discomfort. We discovered severe meibomian gland loss and ocular surface discomfort in patients with PANDO. Meanwhile, we also found that postmenopausal women with PANDO had a higher non-invasive tear meniscus height, which is associated with the degree of nasolacrimal duct obstruction. Thus, non-invasive tear meniscus height may serve as a valuable objective index for assessing the severity of PANDO in clinical settings. In contrast, postmenopausal women with PANDO had lower corneal fluorescein staining and meibomian gland distortion of the upper eyelid scores, which were inversely correlated with the degree of nasolacrimal duct obstruction. Hence, PANDO protects the integrity of the corneal epithelium and prevents meibomian gland distortion, which may be related to the alleviation of tear hyperosmolarity in postmenopausal women.

In 2017, Eom et al. [26] observed that patients with chemotherapy-induced lacrimal duct obstruction exhibited greater meibomian gland loss, similar to our findings, suggesting that primary or secondary lacrimal duct obstruction aggravates damage to the meibomian glands. Some factors that induce lacrimal duct obstruction can also impair the meibomian glands, resulting in meibomian gland loss through a certain mechanism. After controlling for sex and age, which are the greatest risk factors for meibomian gland dysfunction [27], we found that the loss ratio of the upper eyelid was significantly higher in patients with incomplete PANDO than in the normal controls, and the loss ratio of the lower eyelid was significantly higher in patients with incomplete PANDO than in patients with complete PANDO. Thus, meibomian gland loss appears to be more prominent in patients with incomplete PANDO. Interestingly, meibomian gland loss did not bear a positive relation with the degree of nasolacrimal duct obstruction. However, the meibomian gland orifices, secretion expressibility, and meibum quality were not altered significantly in PANDO. Therefore, we postulated that the classical “ductal centric” hypothesis [9, 27] was not applicable to the pathomechanism underlying meibomian gland loss aggravated by PANDO. The ductal centric hypothesis is thought to involve epithelial hyperkeratinization, which causes obstruction of the meibomian gland orifices, meibum stasis, and meibomian gland expansion, resulting in secondary disuse acinar atrophy and meibomian gland loss. Our findings support the “meiotic cell center” hypothesis [28], which involves mechanisms that regulate the renewal and differentiation of meibocytes that may directly affect lipid synthesis, acinar atrophy, and meibum quality, without causing changes in the ductal epithelium. Additionally, the factors that regulate peroxisome proliferator-activated receptor gamma expression and function play a central role in the pathogenesis of meibomian gland dysfunction [29]. Therefore, we speculate that factors regulating the expression and function of peroxisome proliferator-activated receptor gamma in the tears of patients with PANDO would have undergone alteration, which may be attributed to the change in the expression level or emergence of other antagonistic factors. This aspect requires further research, which will be of great clinical significance in the treatment of meibomian gland loss aggravated by PANDO.

Furthermore, the compensatory system in the tear film should be considered. In 2015, Arita et al. [30] demonstrated the existence of a homeostatic system in the tear film, which increases tear secretion to compensate for the deficiency in the oily layer by increasing tear film stability. We found that the non-invasive tear meniscus height and meibomian gland loss were higher in postmenopausal women with PANDO, suggesting that the feedback mechanism induced by the thickened aqueous layer in the tear film may promote meiotic cell apoptosis and meibomian gland loss, thereby decreasing lipid secretion and maintaining tear film homeostasis. Thus, our findings provide novel insights into tear film homeostasis. Meibomian gland loss was more severe in the incomplete PANDO group than in the complete PANDO group, which suggests that the degree of nasolacrimal duct obstruction has differential effects on the feedback mechanism, necessitating further study.

The meibomian gland plays a pivotal role in the maintenance of ocular surface health, while meibomian gland dysfunction is the leading cause of ocular surface diseases [9]. Interestingly, although meibomian gland loss was more severe in postmenopausal women with PANDO, we did not detect significant differences in the meibomian gland orifice, secretion expressibility, meibum quality, or tear film stability. In contrast, PANDO protected corneal epithelial cells. We speculated that the above-mentioned changes in postmenopausal women with PANDO may be attributed to the increase in tear volume that improves liquid insufficiency^11^in postmenopausal women. Another reason could be that tear hypoosmolarity in PANDO improves tear hyperosmolality in postmenopausal women. The occurrence of PANDO is beneficial for maintaining proper tear osmolarity and protecting the corneal epithelium, as evidenced by the results of the ocular surface disease index questionnaire: postmenopausal women with PANDO had few typical symptoms of meibomian gland dysfunction, such as foreign body sensation, burning, or dryness but frequently complained of glare and blurring caused by epiphora. These findings are consistent with those of previous studies that showed greater ocular surface discomfort symptoms in patients with PANDO [5, 31].

In our study, we found that significant morphological changes occur in the meibomian gland in postmenopausal women with PANDO, which are characterized by meibomian gland loss. The mechanism underlying the development of meibomian gland loss aggravated by PANDO showed greater propensity toward the “meiotic cell center” hypothesis. Further research is needed to determine the factors regulating the expression and function of peroxisome proliferator-activated receptor gamma in the tear film of postmenopausal women with PANDO, which would be of great clinical significance for the treatment of meibomian gland loss aggravated by PANDO.

However, our study had some limitations. The sample size of patients with incomplete PANDO was relatively small, probably due to the milder symptoms of ocular surface discomfort. However, interestingly, we found that meibomian gland loss was more serious in postmenopausal women with incomplete PANDO. It is important that these patients receive proper intervention and treatment for PANDO and meibomian gland loss to maintain ocular surface health.

## Conclusion

Our results suggest that PANDO aggravates meibomian gland loss in postmenopausal women, especially in those with incomplete PANDO. Ophthalmologists should identify ocular surface damage in patients with PANDO and provide appropriate treatment. Further studies are necessary to study the changes in tear cytokines in postmenopausal women with PANDO and elucidate the mechanism aggravating meibomian gland loss.

## Data Availability

The data sets used and analyzed during the current study are available from the corresponding author upon reasonable request.

## Abbreviations

NLDO: Nasolacrimal duct obstruction
PANDO: Primary acquired nasolacrimal duct obstruction
